# Absence of oxygen effect on microbial structure and methane production during drying and rewetting events

**DOI:** 10.1038/s41598-022-20448-5

**Published:** 2022-10-04

**Authors:** Tong Liu, Xiaoxiao Li, Sepehr Shakeri Yekta, Annika Björn, Bo-Zhong Mu, Laura Shizue Moriga Masuda, Anna Schnürer, Alex Enrich-Prast

**Affiliations:** 1 Department of Molecular Science, Uppsala BioCenter, Swedish University of Agricultural Science, 750 07 Uppsala, Sweden; 2 Laboratory Medicine Department, Putuo Hospital, Shanghai University of Traditional Chinese Medicine, Shanghai, 200062 People’s Republic of China; 3 Department of Thematic Studies - Environmental Change, Linköping University, 581 83 Linkoping, Sweden; 4 State Key Laboratory of Bioreactor Engineering and Institute of Applied Chemistry, East China University of Science and Technology, Shanghai, 200237 People’s Republic of China; 5 Multiuser Unit for Environmental Analysis, Universidade Federal do Rio de Janeiro – UFRJ, Av. Carlos Chagas Filho, 373, Bloco A, Ilha do Fundão, Rio de Janeiro, RJ CEP 21941-971 Brazil; 6 Biogas Research Center, Linköping University, 581 83 Linköping, Sweden

**Keywords:** Microbial ecology, Archaea

## Abstract

Natural environments with frequent drainage experience drying and rewetting events that impose fluctuations in water availability and oxygen exposure. These relatively dramatic cycles profoundly impact microbial activity in the environment and subsequent emissions of methane and carbon dioxide. In this study, we mimicked drying and rewetting events by submitting methanogenic communities from strictly anaerobic environments (anaerobic digestors) with different phylogenetic structures to consecutive desiccation events under aerobic (air) and anaerobic (nitrogen) conditions followed by rewetting. We showed that methane production quickly recovered after each rewetting, and surprisingly, no significant difference was observed between the effects of the aerobic or anaerobic desiccation events. There was a slight change in the microbial community structure and a decrease in methane production rates after consecutive drying and rewetting, which can be attributed to a depletion of the pool of available organic matter or the inhibition of the methanogenic communities. These observations indicate that in comparison to the drying and rewetting events or oxygen exposure, the initial phylogenetic structure and the organic matter quantity and quality exhibited a stronger influence on the methanogenic communities and overall microbial community responses. These results change the current paradigm of the sensitivity of strict anaerobic microorganisms to oxygen exposure.

## Introduction

Natural environments with frequent drainage are subjected to drying and rewetting events that impose a fluctuation in oxygen (O_2_) exposure caused by drastic changes in the water table. Strict anaerobes are generally believed to have a low tolerance to O_2,_ but they can apply different strategies to cope with such O_2_ exposure, such as by forming resting stages or producing enzymes deactivating reactive oxygen species (ROS)^1^. Additionally, methanogens, although considered O_2_-sensitive microorganisms, have shown to have a recovery capacity to O_2_ exposure promoted by desiccation. Methanogens have often been found in oxidative environments such as rice-paddy anoxic soils that are frequently air drained, and antioxidant genes such as the catalase (KAT) gene have also been detected in their genomes, especially in those of *class-II* methanogens, *e.g.*, *Methanosarcina barkeri*, *Methanobrevibacter arboriphilus*, and *Methanocellales,* also demonstrating aerotolerance^2,3^. Furthermore, the higher resistance to oxidative stress in *Methanocellales*, *Methanomicrobiales* and *Methanosarcinales*, compared to *class-I* methanogens (*e.g.*, *Methanopyrales*, *Methanobacteriales* and *Methanococcales*) has been reported^2^ This resistance might be attributed to their ability to counteract the damage caused by O_2_ and reactive oxygen species (ROS) via enhanced defence/repair mechanisms, such as a more diverse and higher expression level of antioxidant enzymes and the development of a new O_2_/ROS elimination pathway^2^.Thus, it has been recently proposed that the theory concerning O_2_ intolerance in microbiology should be modified, *i.e.*, that some taxa of strict anaerobic methanogens can survive oxic conditions^4^. In addition, their function has been shown to rapidly recover, as evidenced by enhanced organic matter degradation and methane production after the drying of freshwater sediments followed by rewetting^5^. It is unclear what environmental factor might contribute to this recovery, but changes in sedimentary microbial communities were reported after drying and rewetting^6^, which were distinct for sediments of different origins. This observation led to postulations on the potential effect of different organic sources on the microbial response to desiccation^4^, as air drying and rewetting could affect the characteristics of organic matter^7^. Similarly, it has been observed that the mineralization of organic matter was enhanced upon drying and rewetting of soil due to an increased solubilization of organic matter and disruption of soil aggregates^8^. In contrast, Hernandez et al. (2019) observed that in comparison to the drying and rewetting effect, the soil organic matter effect on the microbial community was minor^9^. Conrad (2020) proposed that methanogenic insensitivity to desiccation and O_2_ exposure could be a plausible explanation for these contrasting results and hypothesized that the oxidant resistance of an anaerobic microbial community could be gained or enhanced by their seasonally/periodically drying and rewetting native eco-environments^4^.

However, understanding how the oxidant resistance of an anaerobic microbial community is developed can be limited by studying the native eco-environments since these possible oxidant resistance mechanisms should have already been established in such environments. It is thus reasonable to assume that natural environments subjected to frequent drainage should contain more O_2_-tolerant and/or desiccation-tolerant microorganisms^4^, while anaerobic communities growing in environments that rarely experience aerobic situations should be negatively affected by air-drying and rewetting. Anaerobic digesters (ADs) are engineered systems designed to maintain an anaerobic environment for a relatively long period of time and provide continuous availability of organic matter to maximize organic matter degradation and methanogenesis^10^. Microbial communities in ADs are not exposed to periodic O_2_ (or water loss) and therefore are not expected to be adapted to cope with oxidative stress or desiccation. Thus, the drying and high O_2_ exposure microbial communities experience from ADs should, at least according to the current established knowledge, promote dramatic changes in microbial community structure and functional damage, as shown by their difficulties in resuming methane production. Our results challenged the abovementioned understanding regarding the severe toxicity of O_2_/oxidants to strict anaerobes, revealing some O_2_-tolerant methanogens as well as the yet-to-be-explored mechanism of anaerobe oxidant resistance.

## Materials and methods

### Experimental setup

Anaerobic digester sludge from the (a) Henriksdal wastewater treatment plant (WWTP) (Stockholm, Sweden), (b) Åby biogas plant with food waste (Linköping, Sweden), and (c) Gasum Jordberga with agricultural waste (Linköping, Sweden) were sampled in October 2017 and transported to the laboratory and kept at room temperature (approximately 25 °C) for a few hours. The sludge was then used as both the medium and inoculum source to investigate the effect of drying (under air and N_2_) and rewetting on methane production recovery. Control groups without the drying and rewetting treatment for the three sludges were included. Batch bottles for each type of sludge and treatment were set in triplicate.

In this study, methane production was an indirect measure of methanogenic function and was therefore also interpreted as a microbial function in the studied systems. However, the term methane production was chosen for clarity and to avoid misunderstandings. Methane production performance after one drying and rewetting event (cycle 1) was assessed using an Automatic Methane Potential Test System II (AMPTS II, Bioprocess Control, Sweden). 400 mL of sludge was added to each batch bottle while being flushed with N_2_, leaving 250 mL headspace. All batch bottles were then placed in a thermostat water bath (37 °C) and connected to the AMPTS II system. Mixers were set as stirring in a cycle of 20 min of mixing and 5 min of resting. The produced methane volume was automatically converted to standard temperature and pressure by the software. For air and N_2_ drying, two trays were used for each type of sludge and each treatment. Each tray was supplied with 1.2 L sludge, closed with a lid, connected to an input hose with N_2_ or air and an output hose for the ventilation system in an acclimatized room (37 °C). The hoses were placed inside samples, so air/N_2_ could penetrate deep into the digestates during drying. The drying process lasted around 14 and 20 days for air- and N_2_-dry treatment, respectively, until the sludge samples were completely dried (evaluated by the decrease in TS weight due to water loss). After drying, all batch bottles were filled with certain amount of dried sludge and rewetted with O_2_-free Milli-Q water to reach a 400 mL volume of sludge, and they were then flushed with N_2_ to ensure anaerobic conditions and connected to the AMPTS II as previously described. In parallel, one tray of batch bottles (triplicates) had the same setup except the N_2_ or air-drying treatment was set as a control group. To facilitate subsequent repeated drying-rewetting and incubating operations, cycles 2 and 3 were performed with a similar drying and rewetting process as described in cycle 1 in a traditional batch system. The batch bottle setup and methane content calculation were described previously by Sun et al.^11^, except that 160 mL sludge was added to each 320-mL bottle. For each drying treatment, the bottles were opened and flushed with air or N_2_ in a 37 °C incubation room. For the cycle 2 and 3, sludge samples were completely dry in 5 to 7 days gas flush. There was no stirring during the drying process in all three cycles. Same as cycle 1, the sludge samples were then rewetted with O_2_-free Milli-Q water and reincubated at 37 °C. One tray of batch bottles (triplicate) without the N_2_ or air-drying treatment was set up in parallel as a control group. The produced gas in the control group was released to reset the bottle pressure between each cycle. Approximately 2 mL of sludge was sampled from each batch bottle after each drying-rewetting and incubation event as well as from the original inoculum, and these samples were maintained at −20 °C for later molecular analysis.

### Analytical methods

For cycle 1, total biogas production was determined by using gas meters working based on water displacement. For cycles 2 and 3, the gas pressure in each bottle was regularly measured by a pressure meter (Testo 312, Germany) to quantify the volume of biogas production. Gas samples were collected for headspace methane content analysis by gas chromatography (5880A series, Hewlett Packard, USA). The methane production was normalized to the sludge content of each bottle (*i.e.*, norm. ml g^−1^ digestate) and reported at standard atmospheric pressure and 0 °C. Before and after each treatment, several parameters of the sludge samples were determined. Dissolved organic carbon (DOC) was analysed by a TOC analyser (Shimadzu TOC-V_CPH_, Japan) after the samples were filtered through a 0.45 μm polyethersulfone membrane (USA). VFA concentrations, including acetate, propionate, butyrate, isobutyrate, valerate, and isovalerate, were analysed using a gas chromatograph (6890 Series, Hewlett Packard, USA) according to Jonsson and Borén^12^. The pH was determined using a pH electrode (InfoLab pH 7310, Germany). Student *t-test* and Welch’s ANOVA was performed in R software (version 4.0.2) to analyse the difference of methane production amount and rate across the samples.

### Microbial community analysis

Aliquots of 200 mg in triplicate of each saved sample were used to extract total genomic DNA with the FastDNA spin kit for soil (MP Biomedicals, Santa Ana, CA, USA), as described previously^11^. DNA concentrations were quantified by a Qubit 3.0 Fluorometer (Invitrogen, Thermo Fisher Scientific, Waltham, MA, USA). The 16S rRNA gene sequence library was prepared by polymerase chain reaction (PCR) using the primer pair 515′F(GTGBCAGCMGCC GCG GTAA)/805R(GAC TAC HVGGG TAT CTA ATC C) for the bacterial community and 516F(TGY CAG CCG CCG CGG TAA HACCVGC)/915R(GTG CTC CCC CGC CAA TTC CT) for the archaeal community^13,14^. The detailed PCR procedure and the sequence library preparation steps were described previously^15^. Next-generation amplicon sequencing was performed using Illumina MiSeq technology at the SNP&SEQ Technology Platform of SciLifeLab in Uppsala, Sweden. The raw data were analysed through the open-source bioinformatics pipeline DADA2 (version 1.16) in R software (version 4.0.2)^16^. Forward and reverse sequences were cut to lengths of 259 and 150 bp, respectively, for bacteria with the quality threshold of maxEE = 2 and truncQ = 2 and to 280 and 240 bp for archaea with maxEE = 2 and truncQ = 2, according to in silico calculation by FIGARO^17^. Taxonomic profiles were assigned based on amplicon sequence variants (ASVs) by using the rRNA database SILVA, release 132^18^.

Software STAMP (version 2.1.3)^19^ was used to perform ANOVA and Post-hoc tests for analysing the difference in relative abundance on genus level across samples. Nonmetric multidimensional scaling (NMDS) was applied to assess the dissimilarity of the microbial community among the samples (vegan package in R, permutations = 999). Bacterial community diversity was evaluated using the Hill diversity index ^0^D and ^1^D^20^ in the R package ‘hillR’ (https://CRAN.R-project.org/package=hillR). A combined bacterial and archaeal ASV table with a relative abundance ≥ 0.1% of the total sequence threshold was used to conduct co-occurrence network analysis (RCy3 and igraph package in R). Spearman’s rank correlation was calculated between each pair of ASVs, and *p value*s were corrected by the Benjamini–Hochberg correction to control the false discovery rate upon multiple comparisons^21^. Correlations with significance *p* ≤ 0.001 and coefficients ≥ 0.5 were present in the co-occurrence network. Degree, betweenness, and closeness centrality indices were calculated to evaluate the co-occurrence network structure and potential keystone microorganisms^22^. The co-occurrence network figure was plotted using Cytoscape (version 3.7.2).

## Results and discussion

### Methanogenic production response to drying and O_2_ exposure

To differentiate methanogenic production and microbial community alterations caused by drying from the effects of O_2_ exposure, we dried anaerobic sludge under air and N_2_ atmospheres and rewetted it with O_2_-free water three consecutive times. Sludge samples were retrieved from three different categories of ADs, *i.e.*, sewage sludge, agricultural wastes, and food waste digesters, which are known to potentially have methanogenic communities with distinctively different phylogenetic structures^23,24^. After the drying-rewetting events, the methanogenic production and community structure were evaluated. As expected, methane production rates decreased over time and after each drying-rewetting event, with higher rates from agricultural waste followed by those from food waste and sewage sludge (Table 1). However, the overall trend was constant among all experiments, with a quick recovery of methanogenic production after rewetting for all three drying-rewetting events and with an overall lack of a clear difference between drying with air and drying with N_2_ (Table 1).

**Table 1:** Accumulated methane production of the three types of digestate and the time taken to reach 50, 80 and 100% of the production.

Cycle	Inoculum	Agricultural waste				Food waste				Sewage sludge			
		Days to reach % of the maximum accumulated methane production			Accumulated methane production (norm.mL g^−1^ digestate)	Days to reach % of the maximum accumulated methane production			Accumulated methane production (norm.mL g^−1^ digestate)	Days to reach % of the maximum accumulated methane production			Accumulated methane production (norm.mL g^−1^ digestate)
		50%	80%	100%		50%	80%	100%		50%	80%	100%	
1	Control	8 ± 0	22 ± 0	46 ± 0	7.17 ± 0.24	6 ± 0	16 ± 0	35 ± 0	5.10 ± 0.13	4 ± 1	11 ± 2	27 ± 2	1.39 ± 0.22
	Air dry	15 ± 0	30 ± 2	53 ± 2	7.39 ± 0.21	16 ± 0	19 ± 0	31 ± 1	3.06 ± 0.05*	9 ± 0	15 ± 0	19 ± 2	0.54 ± 0.01*
	N_2_ dry	14 ± 0	28 ± 0	50 ± 0	6.30 ± 0.05*	13 ± 0	18 ± 0	36 ± 0	3.10 ± 0.16*	10 ± 0	14 ± 0	21 ± 1	0.36 ± 0.01*
2	Control	17 ± 0	35 ± 0	48 ± 0	1.95 ± 0.39	20 ± 0	36 ± 0	45 ± 0	0.78 ± 0.07	20 ± 0	32 ± 0	40 ± 0	0.20 ± 0.02
	Air dry	15 ± 0	32 ± 0	42 ± 0	3.70 ± 0.30*	18 ± 0	30 ± 2	45 ± 2	1.73 ± 0.31*	16 ± 0	25 ± 0	40 ± 0	0.44 ± 0.01*
	N_2_ dry	15 ± 1	25 ± 1	45 ± 0	3.33 ± 0.11*	20 ± 0	33 ± 2	40 ± 2	2.19 ± 0.10*	15 ± 0	25 ± 1	40 ± 0	0.73 ± 0.02*
3	Control	18 ± 0	25 ± 0	32 ± 0	0.53 ± 0.07	17 ± 0	31 ± 1	38 ± 1	0.53 ± 0.04	14 ± 1	30 ± 0	42 ± 0	0.15 ± 0.01
	Air dry	13 ± 0	25 ± 1	32 ± 0	0.78 ± 0.22	21 ± 0	28 ± 0	38 ± 0	0.38 ± 0.03*	32 ± 0	40 ± 0	42 ± 0	0.03 ± 0.01*
	N_2_ dry	13 ± 0	25 ± 1	32 ± 2	1.14 ± 0.25*	21 ± 2	28 ± 0	38 ± 0	0.38 ± 0.13	22 ± 0	35 ± 1	42 ± 0	0.15 ± 0.01

One hundred percent of the maximum accumulated methane production (average ± SD, n = 3) was reached when the daily methane production was less than 0.5% of its accumulated value. Student *t-test* was performed to compare the difference in accumulated methane production with two-drying treatments to their control group in each cycle, respectively. Significant differences were marked with “*” when *p* < 0.01.

While the first drying-rewetting event had a negative effect on the methane production of the three types of sludge, the next two drying-rewetting events generally had significant positive effects on the methane production amount and rate compared to those of the control groups (Table 1). For the food waste and sewage sludge samples after the last treatment, methane production performance became similar for both treatments (air and N_2_-dry), possibly due to the depletion of organic matter. The ‘exhausted’ sludge (after one drying event) produced more methane than the undried control, suggesting that drying could enhance the availability of organic matter and favour methanogenic production (Table 1). This result is in line with those of several previous studies on the availability and composition of organic matter, including the evaluation of a range of fractionation procedures, such as sample air-drying followed by rewetting and wet-sieving, recognising that air-drying and rewetting of soil samples affect the characteristics of organic matter (OM)^7^, and increasing OM solubilization and disrupting soil aggregates^8^, which could increase OM availability to microorganisms. Similar results for methane production have also been reported, *i.e.*, an increase in methane production after drying-rewetting in Amazonian oxbow lake sediments^5^ and rain-fed paddy soil from Thailand^25^.

The recovery time of methane production was different between the three different materials and between the first, second, and third drying-rewetting events (Welch’s ANOVA, *p* < 0.01), indicating that the observed different lag phases of methanogenesis were related to the origin of the microbial community and the availability of organic matter. Similar differences have been reported for natural sediments where methanogens have different lag times, depending on their metabolic stage and the presence of electron acceptors other than O_2_^5^. However, in the present study, a surprisingly similar response between the air and N_2_ treatments (except for sewage sludge, which produces much less methane than the other two types of sludge and showed an inconsistent trend) suggests that O_2_ exposure had a limited, if any, influence on the lag phase and recovery of methane production (Table 1).

To assess the effect of drying and rewetting on the solubilization of organic matter, we evaluated the changes in the dissolved organic carbon (DOC) concentrations. The DOC levels varied among the three anaerobic sludge samples, with the highest DOC level occurring in samples from agricultural waste digesters (2526 ± 99 mg/L), followed by those in food waste (1862 ± 18 mg/L) and sewage sludge (293 ± 5 mg/L) digesters. Nevertheless, the DOC levels in the three different sludge types occurred in the same descending order (*i.e.,* agricultural wastes > food waste > sewage sludge) during all drying-rewetting events (Table S1). It is well known that drying can substantially alter the quality and physicochemical characteristics of DOC, *e.g.*, in soil and aquatic sediment samples^26,27^, but information regarding the drying effect on anaerobic sludge is limited to a few studies^28–30^, with no study evaluating the drying effect on DOC in terms of microbes. Nevertheless, Knoop et al. (2018) observed a decrease in DOC during drying in anaerobic digesters operating with municipal organic waste. Their results also showed a significant positive correlation between the DOC level and dissolved Mg, Ca, Ni, and Zn^29^.

Mg, Zn, and Ni concentrations affect microbial enzyme activity^31^, while Ca is involved in the regulation of microbial membrane permeability^32^; thus, a decreased accessibility of these elements might reduce microbial tolerance to drying. As expected, we observed a DOC decrease after each drying and rewetting event across all sludge types independent of N_2_ and air drying. A plausible hypothesis for this decrease is that drying stabilizes micropores due to the cohesion of slightly soluble components and strengthens the aggregates, thus causing a reduction in DOC, as was observed for the soil samples^33,34^. Furthermore, drying can also change the hydrophobicity of particles, and an increase in hydrophobicity in soils can slow the increase in water potential in the following rewetting, thus decreasing the ability of organic matter to redissolve^27,35,36^.

### Archaeal and bacterial community responses to water loss and O_2_ exposure

**Figure 1:**
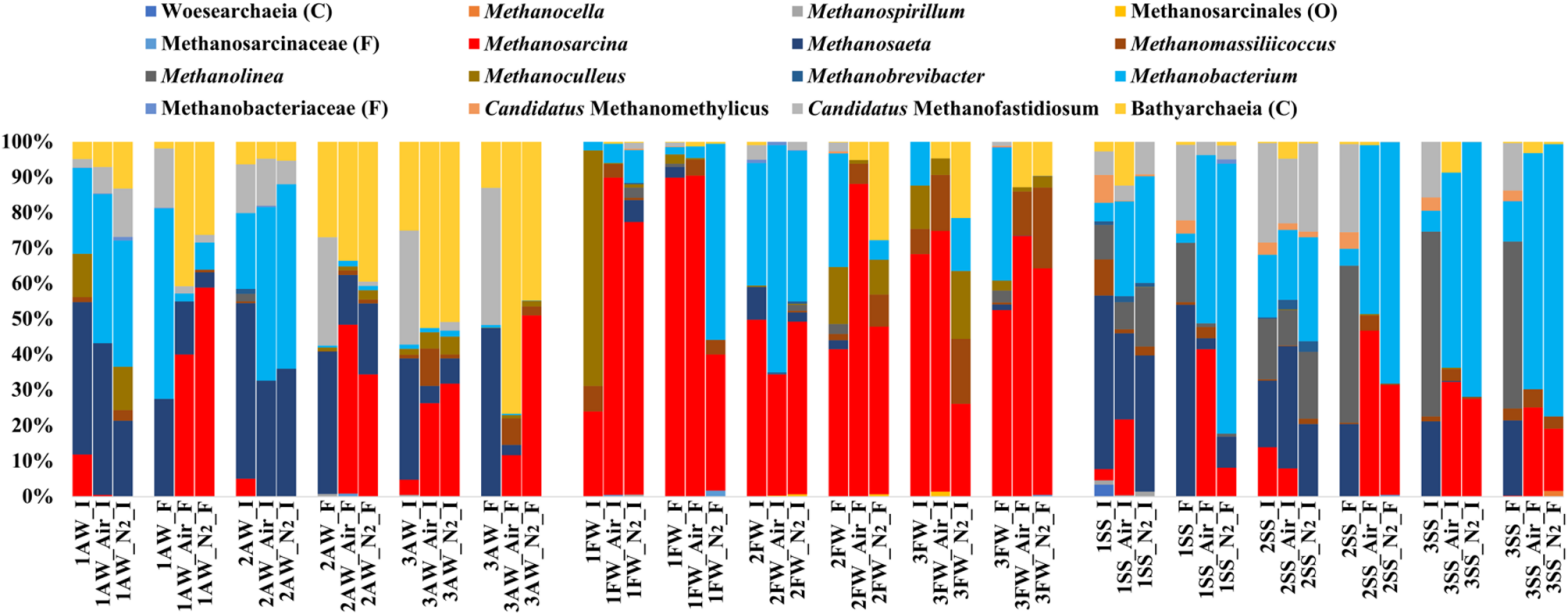
Relative abundance of the archaeal community at the genus level of three types of sludge (*AW* agricultural waste, *FW* food waste, and *SS* sewage sludge) separated after the drying-rewetting (sample name ends with “I”) and incubation events (sample name ends with “F”). From left to right of each figure, blocks corresponding to the first, second, and third drying-rewetting and incubation events (sample names start with 1, 2, and 3, respectively). Where the genus name could not be assigned to the sequences, the closest classified taxonomic level is depicted: class (C), order (O), and family (F).

The archaeal community structures of the three sludge types prior to the experiments (1_AW_I, 1_FW_I, and 1_SS_I) were different, as expected due to their different substrate sources (Fig. 1). Archaeal communities from agricultural waste and sewage sludge digesters were dominated by the obligate acetolactic genus *Methanosaeta* (43 and 48% of the archaeal communities, respectively), while those from the food sludge digester had the highest relative abundance of *Methanoculleus* (66.5% of the archaeal communities), followed by *Methanosarcina* (24.0% of the archaeal communities) (Fig. 1). *Methanosaeta* can only use acetate, and *Methanoculleus* can use H_2_ and formate for methane production, while *Methanosarcina* is very versatile in its substrate range but cannot use formate for methane production^37,38^. The first drying-rewetting event promoted an enrichment of *Methanobacterium* in relation to *Methanosarcina* in the sludge from the agricultural AD. However, the relative abundance of *Methanosarcina* significantly recovered and replaced *Methanobacterium* and *Methanosaeta* (Fig. S1) after the second and third drying-rewetting events, where an unclassified *Bathyarchaeia* archaea also became dominant. Among the archaea genera, *Methanosarcina* had comparatively high relative abundances across all samples from the agricultural and food waste digesters even after the drying and rewetting events, while the relative abundance of *Methanobacterium* largely decreased. In the archaeal community from the sewage sludge digester, *Methanobacterium* prevailed, followed by *Methanosarcina,* after the drying-rewetting events (Fig. 1). Overall, although archaeal communities with different origins developed differently, the drying-rewetting events favoured the establishment of *Methanosarcina* in relation to other methanogens. Interestingly, no clear difference was seen regarding the compositions of the methanogen communities between the air and N_2_-dried treatments.

The most unexpected finding of our study was that O_2_ exposure during the drying and rewetting treatments, scarcely affected the methane production in the later anaerobic incubation step by the three types of sludge, as demonstrated by the similar amounts and rates of methane production after drying with air or N_2_. A slightly longer lag phase for recovery of methane production by the air-dried archaeal community from the food waste in the first rewetting event and sewage sludge in the third rewetting event were exceptions for the whole experiment, whereas similar lag phases were observed for the other treatment conditions (Table 1). These combined results challenge our current understanding of the O_2_ sensitivity of methanogens. In addition, the absence of a clear distinction in the archaeal community structure that would be expected between air- and N_2-_dried anaerobic sludge suggests that the O_2_ resistance capacity was native even for the methanogenic communities living in strict anaerobic environments. A few groups of methanogens, in this study represented by members of *Methanosarcina*, showed a higher stress tolerance than that of other methanogens of the archaeal community (Fig. 1). In ADs, compared to other methanogens, *Methanosarcina* has often been identified as the dominant methanogen due to its high adaptability to variable digester operating conditions, such as low pH and high ammonia and salt content^39^. A dominance of *Methanosarcina* also occurs in natural ecosystems, for instance, in Amazonian oxbow lake sediments that undergo periodic rainy and dry seasons^5^ and biological soil crusts of arid regions experiencing dry and oxic conditions^40^. A recent study grouped *Methanosarcina* in *class-II* among 40 other methanogens regarding oxidant tolerance according to their genome sequences. Furthermore, the study indicated that this high-oxidant tolerance might be attributed to a more diverse and higher expression level of antioxidant enzymes and the development of a new O_2_/ROS elimination pathway compared to those of *class-I* methanogens^2^. *Methanosarcina* is also flexible in its substrate utilization and grows faster than *Methanosaeta*^41^, which contribute to its high tolerance under stressed conditions. Furthermore, the high stress tolerance of *Methanosarcina* may be further enhanced when harboured in a community instead of as a pure culture^42,43^. The drying event, however, did not explain the decrease in *Methanoculleus* (*class-II*) in the food waste sludge, considering that *Methanoculleus* has also been shown to be predominant in dry and oxic soils^40^. A change in the organic matter characteristics after the drying and rewetting events leading to an increase in the availability of acetate in relation to H_2_ or formate is a possible explanation. This scenario would have favoured the revival of acetolactic *Methanosarcina* instead of formate- and hydrogenotrophic *Methanoculleus* in the agricultural and food waste sludge. At the comparably low level of organic matter in the sewage sludge, this effect might have been limited.

**Figure 2:**
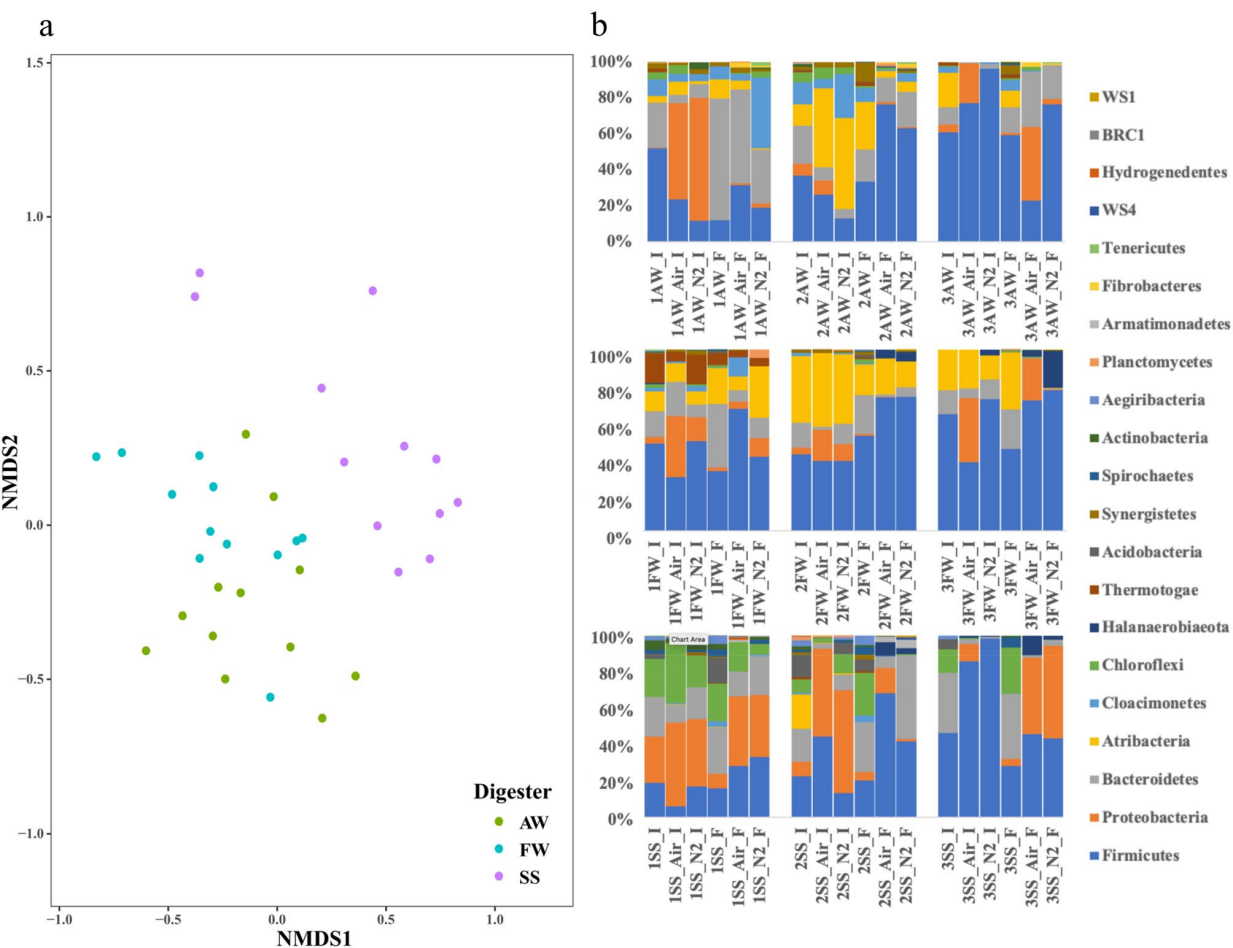
Bacterial community structure of the three types of sludge (AW: agricultural waste, FW: food waste, and SS: sewage sludge). (**a**) Nonmetric multidimensional scaling (NMDS) analysis based on ASV read counts displaying bacterial community distribution coloured by sludge types. (**b**) Relative abundance of the bacterial community at the phylum level of three types of sludge separated after the drying-rewetting (sample name end with “I”) and incubation events (sample name end with “F”). From top to bottom, the bar plots correspond to the agricultural waste, food waste, and sewage sludges. From left to right, the bar plots correspond to the first, second, and third drying-rewetting and incubation events (sample names start with 1, 2, and 3, respectively). Bacteria with relative abundances ≥ 1.0% in at least one sample are depicted.

The bacterial community structure of the three sludge types had relatively similar compositions but with varying relative abundances (Fig. 2a and b). The phylum Firmicutes accounted for 52 and 48% of the total bacteria from the agricultural and food waste digesters, respectively, followed by Bacteroidetes (25 and 14% of bacteria, respectively). The phyla Cloacimonetes, Chloroflexi, Atribacteria, and Thermotogae were also dominant in the agricultural and food waste digesters, with a few differences, including a higher relative abundance of Thermotogae (16%) in the food waste digester than in the agricultural waste digester and the presence of Synergistets in the agricultural waste digester. The bacterial community from the sewage sludge digester was dominated by the phyla Proteobacteria (25%), Bacteroidetes (22%), Chloroflexi (21%) and Firmicutes (19%). Actinobacteria (6%), Acidobacteria (3%), Aegiribacteria (2%) and Spirochaetes (2%) were also present at low abundances, while they were absent in the samples from agricultural and food waste digesters (Fig. 2b).

The first drying-rewetting event altered the structure of the three bacterial communities, with a one order of magnitude substantial increase in the relative abundance of Proteobacteria from < 1 to 53% (air-dried) and 68% (N_2_-dried) in agricultural waste sludge; from 4% to up to 34% (air-dried) and 13% (N_2_-dried) for food waste sludge; and from 25% to up to 46% (air-dried) and 37% (N_2_-dried) for sewage sludge (Fig. 2b). However, the relative abundance of Proteobacteria dropped (< 10%) after the first incubation event, where Firmicutes, particularly in the agricultural and food waste digestate samples, dominated the bacterial community. The same pattern, *i.e.*, alternating dominance between Proteobacteria and Firmicutes during the drying-rewetting and incubating events, was repeated in the second and third rounds of treatments, and at the end, they outcompeted most of the other bacteria [also shown as decreased population diversity indices (Table S2)]. At the genus level, this process occurred as a unified increase in the relative abundance of *Hydrogenispora* and *Alkaliphilus* within the phylum Firmicutes (Fig. S2), while for Proteobacteria, the increase in relative abundance differed somewhat according to the origin of the microbial communities, represented by the genus *Pusillimonas* in the agricultural and food waste samples and an unclassified member of the family Burkholderiaceae in the sewage sludge samples (Fig. S3). In addition, the family Halobacteroidaceae, also belonging to the phylum Proteobacteria, increased at the end of the experiment in the food waste and sewage sludge samples. The genus *Hydrogenispora* can ferment various sugars to produce hydrogen and acetate, while *Alkaliphilus* can ferment sugars to acetate and formate^44,45^. Members of the family Burkholderiaceae and Halobacteroidaceae have also been shown to ferment various sugars^46,47^, while the genus *Pusillimonas* can use acetate as a carbon source and potentially compete with methanogens^48^. The increase in the relative abundance of *Pusillimonas* after the drying-rewetting events, especially later for the air-dried samples, suggests that acetolactic bacteria might have competed temporally with acetoclastic methanogens (in our case *Methanosarcina)* for acetate, especially when the latter was inhibited.

Microbial community dynamics under dry and oxic stress in ADs have garnered limited interest, and most studies have focused on the responses in natural environments such as soil and lake sediments where an overall trend of increasing gram-positive phyla (*e.g.,* Firmicutes) in relation to gram-negative phyla (*e.g.,* Proteobacteria and Bacteroidetes) has been reported^5,49^. The tolerance of bacteria to drying/drought stress has also been relatively well studied. The physiological characteristics influencing tolerance mainly include the thickness of cell walls and the ability of sporulation^49^. Thus, it is not surprising that *Hydrogenispora* and *Alkaliphilus* (belonging to Firmicutes), both spore-forming and gram-positive bacteria, exhibited an overall higher stress tolerance than that of Bacteroidetes (represented by order Bacteroidales, mostly gram-negative) in our study^44,50–55^. However, these arguments do not explain the increase in the abundance of the phylum Proteobacteria, represented by the genera *Pusillimonas*, *Pseudomonas*, and *Nitrincola* and the families Burkholderiaceae and Halobacteroidaceae in the present study, which are all gram-negative and nonspore-forming bacteria, with the exception of a few species in Halobacteroidaceae that can produce endospores^46,47,56–58^. Thus, there must be other bacterial strategies that counter drying effects. One possible explanation could be the ability of these bacteria to produce and accumulate osmolytes, contributing to the maintenance of cellular turgor and protection of macromolecular structure in microorganisms^59^. Another possibility could be an established network between different microbial groups, *i.e.*, a beneficial cooperative interaction in microbial communities might potentially enhance their environmental stress resistance^60^.

### Co-occurrence network analysis of the microbial community

**Figure 3:**
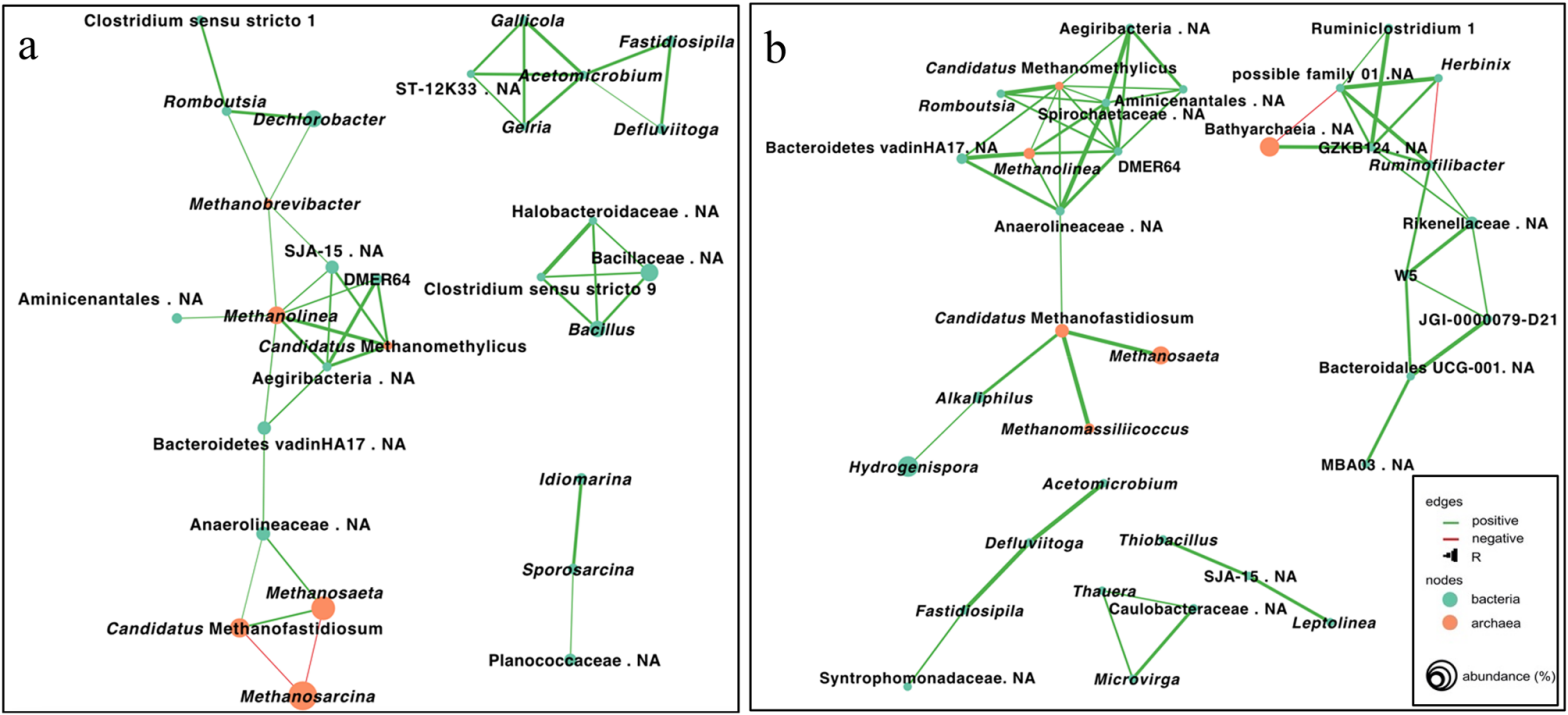
Co-occurrence network analysis based on the correlation of the relative abundance of amplicon sequence variant (ASV) reads for the microbial profiles at the genus level or the closest classified taxonomic level. (**a**) after the drying-rewetting and (**b**) incubation events. Bacterial and archaeal groups are shown by nodes in green and orange, respectively. Each edge represents significant correlations between pairs of nodes (*p* ≤ 0.001), where positive and negative correlations are coloured in green and red, respectively. The thickness of the edge is proportional to the R value of the correlation (R ≥ 0.5).

To assess whether drying-rewetting stress might affect cooperative interactions in microbial communities, we conducted a co-occurrence network analysis on microbial communities after the drying-rewetting and incubation events. Figure 3 displays signification correlations (*p* ≤ 0.001, R ≥ 0.5) in a microbial network constituted by archaea and bacteria containing at least three nodes (≥ 0.1% in relative abundance) at the genus level based on their degree, betweenness, and closeness centrality. The network pattern clearly differed between the drying-rewetting and recovery/incubation events (Fig. 3a and b). A slightly less clustered and overall lower R value occurred for the drying-rewetting group (Fig. 3a) than for the recovery/incubation group (Fig. 3b). An unclassified genus belonging to the phylum Aegiribacteria and genus *Methanolinea* was positively linked to each other and showed a relatively high degree of centrality in both networks (Fig. 3, Tables S3 and S4). These taxa, however, occurred with low relative abundances in the samples (Fig. 2). For the drying- and rewetting-treated samples (Fig. 3a), Aegiribacteria was also positively correlated with the genus *Candidatus* Methanofastidiosum via Bacteriodes vadinHA17 and Anaerolineaceae, which was further correlated with a subcluster of methanogens, including *Candidatus* Methanomethylicus and *Methanosaeta*; however, it was negatively correlated with *Methanosarcina*. Interestingly, the competition relationship between *Methanosarcina* and the other two methanogens was absent and replaced by a positive interaction between two bacterial genera *Hydrogenispora* and *Alkaliphilus,* particularly increasing after the incubation stage (Fig. 3b).

In general, the network analysis suggested an overall weakened network of interactions between microorganisms after the drying-rewetting events, while a new positive correlation was identified after incubation events among the increased bacteria *Hydrogenispora* and *Alkaliphilus*, together with a subcluster of methanogens. *Hydrogenispora* and *Alkaliphilus* have also both been found in rice paddies, another environment exposed to periodical drying and rewetting events^61,62^. Since *Hydrogenispora* and *Alkaliphilus* are both identified as fermentative bacteria, producing acetate as well as hydrogen (*Hydrogenispora)* and formate (*Alkaliphilus)*^44,45^, our observations implied that their increase in relative abundance could potentially serve as new substrate suppliers for both formate-dependent, hydrogenotrophic, and acetotrophic methanogens. The correlations between Aegiribacteria and *Methanolinea* have yet to be explored, except for a recent study reporting their cooccurrence in anaerobic digesters with high sulfide content^63^. Moreover, Aegiribacteria and *Methanolinea* showed a relatively high degree of centrality, closeness and connectance in both the drying-rewetting and recovering groups, while their betweenness drastically decreased after the incubation stage (Table S4), suggesting that they might act as keystone species in methanogenic function recovery according to Berry and Widder^22^.

### Future perspective

In contrast to the current established knowledge regarding the severe toxicity of O_2_/oxidants to methanogens, our observations indicated unexpectedly similar methanogen community structure and methane production recovery after drying/wetting with and without the presence of O_2_. It is also not clear whether drying promotes the anaerobic microbial community in establishing their strategy to resist O_2_/oxidants. Therefore, future research should carefully examine genes that are regulated in methanogens under these conditions to further reveal their self-protecting mechanisms under O_2_/oxidants or drying stress. Our study showed that the original characteristics of the AD sludge, including DOC content and the composition of microbial communities, seemed to be key factors determining drying and rewetting tolerance, as revealed by methane production. Repeatedly recovered methane production after drying and rewetting events implies that microorganisms may form new metabolic interaction networks leading to more stress-tolerant communities.

## Supplementary Information

Supplementary Figure S1.

Supplementary Figures S2 and S3.

Supplementary Table S1.

Supplementary Table S2.

Supplementary Table 3.

Supplementary Table S4.

## Data Availability

The raw DNA sequencing data obtained in the current study are available in the National Center for Biotechnology Information database (NCBI) under accession number PRJNA736312, BioSample: SAMN19641899 (Bacteria), SAMN19643011 (Archaea).

## References

[1] Madigan, M. T., Bender, K. S., Buckley, D. H., Sattley, W. M. & Stahl, D. A. (Pearson Benjamin Cummings, San Francisco, CA94111, 2021).

[2] Lyu Z, Lu Y (2018). Metabolic shift at the class level sheds light on adaptation of methanogens to oxidative environments. ISME J..

[3] Brioukhanov AL, Netrusov AI, Eggen RIL (2006). The catalase and superoxide dismutase genes are transcriptionally up-regulated upon oxidative stress in the strictly anaerobic archaeon Methanosarcina barkeri. Microbiology.

[4] Conrad R (2020). Methane production in soil environments-anaerobic biogeochemistry and microbial life between flooding and desiccation. Microorganisms.

[5] Conrad R (2014). Response of the methanogenic microbial communities in Amazonian oxbow lake sediments to desiccation stress. Environ. Microbiol..

[6] Ji Y (2016). Structure and function of methanogenic microbial communities in sediments of Amazonian lakes with different water types. Environ. Microbiol..

[7] Kaiser M, Kleber M, Berhe AA (2015). How air-drying and rewetting modify soil organic matter characteristics: An assessment to improve data interpretation and inference. Soil Biol. Biochem..

[8] Wu J, Brookes PC (2005). The proportional mineralisation of microbial biomass and organic matter caused by air-drying and rewetting of a grassland soil. Soil Biol. Biochem..

[9] Hernández M (2019). Structure, function and resilience to desiccation of methanogenic microbial communities in temporarily inundated soils of the Amazon rainforest (Cunia Reserve, Rondonia). Environ. Microbiol..

[10] Schnürer, A., Bohn, I. & Moestedt, J. in *Hydrocarbon and Lipid Microbiology Protocols: Bioproducts, Biofuels, Biocatalysts and Facilitating Tools* (eds Terry J. McGenity, Kenneth N. Timmis, & Balbina Nogales) 171–200 (Springer Berlin Heidelberg, 2017).

[11] Sun L, Liu T, Müller B, Schnürer A (2016). The microbial community structure in industrial biogas plants influences the degradation rate of straw and cellulose in batch tests. Biotechnol. Biofuels.

[12] Jonsson S, Borén H (2002). Analysis of mono- and diesters of o-phthalic acid by solid-phase extractions with polystyrene–divinylbenzene-based polymers. J. Chromatogr. A.

[13] Hugerth LW (2014). DegePrime, a program for degenerate primer design for broad-taxonomic-range PCR in microbial ecology studies. Appl. Environ. Microbiol..

[14] Takai K, Horikoshi K (2000). Rapid detection and quantification of members of the archaeal community by quantitative PCR using fluorogenic probes. Appl. Environ. Microbiol..

[15] Liu T, Schnürer A, Björkmalm J, Willquist K, Kreuger E (2020). Diversity and abundance of microbial communities in UASB reactors during methane production from hydrolyzed wheat straw and lucerne. Microorganisms.

[16] Callahan BJ (2016). DADA2: High-resolution sample inference from Illumina amplicon data. Nat. Methods.

[17] Weinstein MM, Prem A, Jin M, Tang S, Bhasin JM (2019). FIGARO: An efficient and objective tool for optimizing microbiome rRNA gene trimming parameters. bioRxiv.

[18] Quast C (2013). The SILVA ribosomal RNA gene database project: Improved data processing and web-based tools. Nucl. Acids Res..

[19] Parks DH, Tyson GW, Hugenholtz P, Beiko RG (2014). STAMP: Statistical analysis of taxonomic and functional profiles. Bioinformatics.

[20] Lucas R (2017). A critical evaluation of ecological indices for the comparative analysis of microbial communities based on molecular datasets. FEMS Microbiol. Ecol..

[21] Haynes, W. in *Encyclopedia of Systems Biology* (eds Werner Dubitzky, Olaf Wolkenhauer, Kwang-Hyun Cho, & Hiroki Yokota) 78–78 (Springer New York, 2013).

[22] Berry D, Widder S (2014). Deciphering microbial interactions and detecting keystone species with co-occurrence networks. Front. Microbiol..

[23] Liu T, Sun L, Schnürer BMA (2017). Importance of inoculum source and initial community structure for biogas production from agricultural substrates. Bioresour. Technol..

[24] Sundberg C (2013). 454 pyrosequencing analyses of bacterial and archaeal richness in 21 full-scale biogas digesters. FEMS Microbiol. Ecol..

[25] Reim A (2017). Response of methanogenic microbial communities to desiccation stress in flooded and rain-fed paddy soil from Thailand. Front. Microbiol..

[26] del Campo R, Gómez R, Singer G (2019). Dry phase conditions prime wet-phase dissolved organic matter dynamics in intermittent rivers. Limnol. Oceanogr..

[27] Borken W, Matzner E (2009). Reappraisal of drying and wetting effects on C and N mineralization and fluxes in soils. Glob. Change Biol..

[28] Awiszus S, Meissner K, Reyer S, Müller J (2018). Ammonia and methane emissions during drying of dewatered biogas digestate in a two-belt conveyor dryer. Bioresour. Technol..

[29] Knoop C, Dornack C, Raab T (2018). Effect of drying, composting and subsequent impurity removal by sieving on the properties of digestates from municipal organic waste. Waste Manage..

[30] Salamat R, Scaar H, Weigler F, Berg W, Mellmann J (2020). Drying of biogas digestate: A review with a focus on available drying techniques, drying kinetics, and gaseous emission behavior. Dry. Technol..

[31] Šafarič L (2020). Effect of cobalt, nickel, and selenium/tungsten deficiency on mesophilic anaerobic digestion of chemically defined soluble organic compounds. Microorganisms.

[32] Matheri, A. N., Belaid, M., Seodigeng, T. & Ngila, J. C. In *Proceedings of the World Congress on Engineering*, London, UK.

[33] Kemper W, Rosenau R (1984). Soil cohesion as affected by time and water content. Soil Sci. Soc. Am. J..

[34] Peng X, Horn R (2005). Modeling Soil shrinkage curve across a wide range of soil types. Soil Sci. Soc. Am. J..

[35] Halverson LJ, Jones TM, Firestone MK (2000). Release of intracellular solutes by four soil bacteria exposed to dilution stress. Soil Sci. Soc. Am. J..

[36] Schimel J, Balser TC, Wallenstein M (2007). Microbial stress-response physiology and its implications for ecosystem function. Ecology.

[37] Smith KS, Ingram-Smith C (2007). Methanosaeta, the forgotten methanogen?. Trends Microbiol..

[38] Thauer RK, Kaster A-K, Seedorf H, Buckel W, Hedderich R (2012). Methanogenic archaea: Ecologically relevant differences in energy conservation. Nat. Rev. Microbiol..

[39] De Vrieze J, Hennebel T, Boon N, Verstraete W (2012). Methanosarcina: The rediscovered methanogen for heavy duty biomethanation. Bioresour. Technol..

[40] Angel R, Claus P, Conrad R (2012). Methanogenic archaea are globally ubiquitous in aerated soils and become active under wet anoxic conditions. ISME J..

[41] Schnürer A (2012). Biogas production: Microbiology and technology. Adv Biochem Eng Biotechnol..

[42] Kato MT, Field JA, Lettinga G (1993). High tolerance of methanogens in granular sludge to oxygen. Biotechnol. Bioeng..

[43] Kiener A, Leisinger T (1983). Oxygen sensitivity of methanogenic bacteria. Syst. Appl. Microbiol..

[44] Liu Y, Qiao J-T, Yuan X-Z, Guo R-B, Qiu Y-L (2014). Hydrogenispora ethanolica gen. nov., sp. Nov., an anaerobic carbohydrate-fermenting bacterium from anaerobic sludge. Int. J. Syst. Evolut. Microbiol..

[45] Wu X-Y (2010). Alkaliphilus halophilus sp. Nov., a strictly anaerobic and halophilic bacterium isolated from a saline lake, and emended description of the genus Alkaliphilus. Int. J. Syst. Evolut. Microbiol..

[46] Coenye, T. in *The Prokaryotes: Alphaproteobacteria and Betaproteobacteria* (eds Eugene Rosenberg et al.) 759–776 (Springer Berlin Heidelberg, 2014).

[47] Oren, A. in *Bergey's Manual of Systematics of Archaea and Bacteria* 1–4.

[48] Stolz A, Bürger S, Kuhm A, Kämpfer P, Busse H-J (2005). Pusillimonas noertemannii gen. nov., sp. nov., a new member of the family Alcaligenaceae that degrades substituted salicylates. Int. J. Syst. Evolut. Microbiol..

[49] Naylor D, Coleman-Derr D (2018). Drought stress and root-associated bacterial communities. Front. Plant. Sci..

[50] Acosta-Martínez V (2014). Predominant bacterial and fungal assemblages in agricultural soils during a record drought/heat wave and linkages to enzyme activities of biogeochemical cycling. Appl. Soil. Ecol..

[51] Bouskill NJ (2013). Pre-exposure to drought increases the resistance of tropical forest soil bacterial communities to extended drought. ISME J..

[52] Chodak M, Gołębiewski M, Morawska-Płoskonka J, Kuduk K, Niklińska M (2015). Soil chemical properties affect the reaction of forest soil bacteria to drought and rewetting stress. Ann. Microbiol..

[53] Curiel Yuste J (2014). Strong functional stability of soil microbial communities under semiarid Mediterranean conditions and subjected to long-term shifts in baseline precipitation. Soil Biol. Biochem..

[54] Hartmann M (2017). A decade of irrigation transforms the soil microbiome of a semi-arid pine forest. Mol. Ecol..

[55] Zakharyuk A (2017). Alkaliphilus namsaraevii sp. nov., an alkaliphilic iron- and sulfur-reducing bacterium isolated from a steppe soda lake. Int. J. Syst. Evolut. Microbiol..

[56] Jin L (2017). Pusillimonas caeni sp. nov., isolated from a sludge sample of a biofilm reactor. Antonie Van Leeuwenhoek.

[57] Joshi A (2020). Nitrincola tapanii sp. nov., a novel alkaliphilic bacterium from An Indian Soda Lake. Int. J. Syst. Evol. Microbiol..

[58] Palleroni, N. J. Pseudomonas. *Bergey's Manual of Systematics of Archaea and Bacteria.*10.1002/9781118960608.gbm01210 (2015).

[59] Welsh DT (2000). Ecological significance of compatible solute accumulation by micro-organisms: From single cells to global climate. FEMS Microbiol. Rev..

[60] Morris BEL, Henneberger R, Huber H, Moissl-Eichinger C (2013). Microbial syntrophy: Interaction for the common good. FEMS Microbiol. Rev..

[61] Li H, Peng J, Weber KA, Zhu Y (2011). Phylogenetic diversity of Fe(III)-reducing microorganisms in rice paddy soil: Enrichment cultures with different short-chain fatty acids as electron donors. J. Soils Sedim..

[62] Yi X-Y (2019). Coupling metabolisms of arsenic and iron with humic substances through microorganisms in paddy soil. J. Hazard. Mater..

[63] Shakeri Yekta S (2021). Effluent solids recirculation to municipal sludge digesters enhances long-chain fatty acids degradation capacity. Biotechnol. Biofuels.

