# Supplementary material for: Absence of oxygen effect on microbial structure and methane production during drying and rewetting events
Source: Sci Rep. 2022 Oct 4;12:16570. doi: 10.1038/s41598-022-20448-5 (PMC9532411; doi:10.1038/s41598-022-20448-5)
Supplement: Supplementary file 1 — Supplementary Figure S1. [file 41598_2022_20448_MOESM1_ESM.docx]

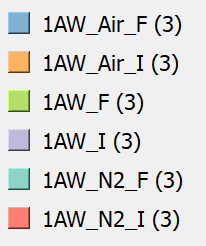

**Figure S1.** **(a).** boxplot for comparing the relative abundance of genus *Methanosarcina* in agriculture waste samples (n=3) in the first drying and rewetting cycles. **(b).** Post-hoc test with statistic method ANOVA and Tukey-Kramer for comparing the relative abundance of genus *Methanosarcina* in agriculture waste samples (n=3) in the first drying and rewetting cycles.
